# A284 DIAGNOSTIC ACCURACY OF THE ABDOMINAL X-RAY IN CHILDHOOD CONSTIPATION: A SYSTEMATIC REVIEW OF THE LITERATURE

**DOI:** 10.1093/jcag/gwac036.284

**Published:** 2023-03-07

**Authors:** D Avelar Rodriguez, G Dahlwi, M Gould, P Marcon, M Benninga

**Affiliations:** 1 The Hospital for Sick Children, Toronto, Canada; 2 Emma Children’s Hospital, Amsterdam, Netherlands

## Abstract

**Background:**

Childhood constipation is a relatively common condition in pediatrics, with a global pooled prevalence of 9.5%. The most common form of constipation in children is functional in nature, a clinical diagnosis defined by the Rome IV criteria.

Although available data discourage the use of the abdominal x-ray (AXR) for the evaluation of constipation in children due to its limited value, radiation exposure, as well as its possible misleading nature, a significant number of clinicians continue to use AXRs in children with constipation.

Given that it has been 10 years since the last systematic review on this topic was published, an updated systematic review is now warranted.

**Purpose:**

To evaluate the diagnostic accuracy of the abdominal x-ray in children with functional constipation (FC).

**Method:**

The protocol was registered (PROSPERO ID: CRD42022301833) and the Preferred Reporting Items for Systematic Reviews and Meta-Analyses (PRISMA) guidelines were followed.

**Eligibility criteria**

Study population included children and adolescents aged 2 to 18 years with a clinical diagnosis of FC in whom AXR was performed. The radiographic parameters that were used to evaluate the AXR had to be specified by the authors. Exclusion criteria included children with organic causes of constipation and children younger than two years.

**Information sources, search strategy and selection criteria**

A reference librarian searched the Medline, Embase and Scopus databases from 2012 (year of last systematic review) to May 2022. The search was limited to English only. In addition, reference lists from selected papers were screened for relevant articles.

**Data collection**

Two authors independently screened eligible articles. Disagreements between investigators were resolved by consensus with a third investigator. Extracted data was inputted into a structured collection sheet by two authors working independently.

**Risk of bias assessment and applicability concerns**

To assess the risk of bias and applicability concerns, two investigators working independently applied six of the QUADAS-2 items to each of the included articles.

**Result(s):**

The search identified a total of 1,125 articles, of which only three were included in the final qualitative analysis. A quantitative analysis was not conducted due to clinical and methodologic heterogeneity. Two studies were conducted in the USA (case-control studies) and one study was conducted in Iran (cohort study), involving a total of 416 children. All three studies used different radiographic parameters to define constipation. The two case-control studies were deemed unclear-to-low risk of bias, while the cohort study was considered unclear-to-high risk of bias. **Figure 1** shows the study findings.

**Image:**

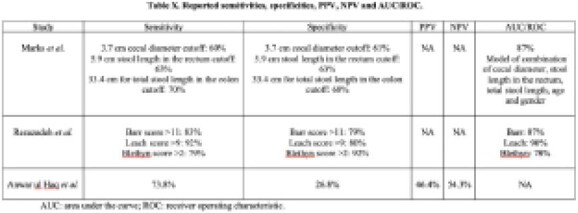

**Conclusion(s):**

There is insufficient evidence to support the use of AXR for the diagnostic workup of FC. More methodologically rigorous studies are needed in order to determine the utility of the AXR in FC. The diagnosis of FC should be based on history and clinical findings.

**Please acknowledge all funding agencies by checking the applicable boxes below:**

None

**Disclosure of Interest:**

None Declared

OBESITY, METABOLIM & NUTRITION

